# Outer Membrane Lipoprotein Lip40 Modulates Adherence, Colonization, and Virulence of *Actinobacillus pleuropneumoniae*

**DOI:** 10.3389/fmicb.2018.01472

**Published:** 2018-07-03

**Authors:** Jinlin Liu, Yurou Cao, Lulu Gao, Li Zhang, Siying Gong, Jihong Yang, Haobin Zhao, Dengfu Yang, Jin Zhao, Jianzhong Meng, Qishuang Gao, Chao Qi

**Affiliations:** ^1^Hubei Key Laboratory of Genetic Regulation and Integrative Biology, College of Life Sciences, Central China Normal University, Wuhan, China; ^2^Lichuan Municipal Bureau of Animal Husbandry and Veterinary Medicine, Lichuan, China; ^3^Department of Animal Biotechnology and Cell Engineering, Wuhan Institute of Animal Husbandry and Veterinary Sciences, Wuhan Academy of Agricultural Sciences, Wuhan, China

**Keywords:** *Actinobacillus pleuropneumoniae*, Lip40, adherence, colonization, virulence

## Abstract

Bacterial lipoproteins are a set of membrane proteins with various functions; many of which are virulence factors of pathogenic bacteria. In the present study, we investigated the role of an outer membrane lipoprotein Lip40 in the pathogenesis of *Actinobacillus pleuropneumoniae*. A mutant strain (Δ*lip40*) lacking Lip40 and a complemented strain (CΔ*lip40*) were constructed. Δ*lip40* exhibited reduced adherence to the St. Jude porcine lung cells. The ability of the Δ*lip40* mutant to colonize the mouse lung tissues was significantly impaired compared to that of the wild type and complementation strains. Furthermore, an infection assay revealed that pigs infected with Δ*lip40* showed fewer clinical signs and lung lesions, indicating that Lip40 contributed to the development of porcine pleuropneumonia. Collectively, our data suggest that Lip40 is involved in the virulence of *A. pleuropneumoniae*.

## Introduction

*Actinobacillus pleuropneumoniae* is a Gram-negative bacterium that is the causative agent of porcine pleuropneumonia, which is often characterized by hemorrhagic, necrotic pneumonia and fibrinous pleuropneumonia and associated with large economic losses worldwide (Fenwick and Henry, 1994). A total of 16 serovars of *A. pleuropneumoniae* have been classified based on the antigenicity of capsular polysaccharide (CPS) and/or lipopolysaccharide (LPS) (Sárközi et al., 2015). All these serovars of *A. pleuropneumoniae* are able to cause the disease. Although the pathogenesis of *A. pleuropneumoniae* infection is not fully understood, colonization, immune evasion and damage to the host are known as the three basic stages (Bossé et al., 2002). Several factors are involved in these processes, such as exotoxins, CPS, LPS, adhesins, proteases, outer membrane proteins and transcriptional regulators (Chiers et al., 2010).

Bacterial lipoproteins are a set of multifunctional membrane proteins that are characterized by lipid modification at the conserved *N*-terminal cysteine residue (Okuda and Tokuda, 2011). Bacterial lipoproteins play various roles in cellular processes, such as nutrient transport, cell division, cellular structure maintenance, and signal transduction (Okuda and Tokuda, 2011). Lipoproteins are important in many aspects of virulence of pathogenic bacteria, including adhesion, invasion, colonization, immune evasion, and immunomodulation (Kovacs-Simon et al., 2011). Although many lipoproteins are predicted in *A. pleuropneumoniae* (Chung et al., 2007; Hu et al., 2015), the functions of these putative lipoproteins in the physiological and pathogenic activities have rarely been investigated.

A putative lipoprotein, Lip40, was identified in our previous study (Hu et al., 2015). It is located on the outer membrane and is stress responsive and immunoprotective. We assumed that Lip40 might be linked to the pathogenicity of *A. pleuropneumoniae*. In this study, we constructed a Lip40 knockout mutant (Δ*lip40*) and the corresponding complementation strain (CΔ*lip40*) based on *A. pleuropneumoniae* SLW01 (serovar 1). Here, we examined the cell adherence, colonization, and virulence of these *A. pleuropneumoniae* strains.

## Materials and Methods

### Bacteria Strains, Plasmids, and Primers

The bacterial strains, plasmids and primers used in this study are listed in **Table 1**. *A. pleuropneumoniae* strains were cultured on trypic soy agar (TSA; Dickinson and Company, Franklin Lakes, NJ, United States) or in tryptic soy broth (TSB), supplemented with 10 μg/ml nicotinamide adenine dinucleotide (NAD^+^; Sigma, St. Louis, MO, United States) and 5% fetal calf serum (Gibco BRL, Grand Island, NY, United States). During the construction of *A. pleuropneumoniae lip40* mutants, 5 μg/ml chloramphenicol was added for the selection of single crossover mutants, 5% sucrose (m/v) was supplemented for double crossover mutant selection, and 2 μg/ml chloramphenicol was used for complementation strain selection. *Escherichia coli* β2155 transformed with transconjugation plasmid was grown on Luria–Bertani (LB) agar (Oxoid, Basingstoke, Hants, United Kingdom) or in LB broth with 50 μg/ml diaminopimelic acid (Sigma) and ampicillin (100 μg/ml).

**Table 1:** Bacterial strains, plasmids, and primers used in this study.

Strains, plasmids, and primers	Relevant characteristics	Sources
**A. pleuropneumoniae**		
SLW01	Serovar 1	Lin et al., 2007
Δ*lip40*	*A. pleuropneumoniae* SLW01 *lip40*-deletion mutant	This work
CΔ*lip40*	*A. pleuropneumoniae* strain Δ*lip40* containing complementation plasmid pJFF-*lip40*	This work
*E. coli*		
DH5α	Cloning vehicle: *supE44* aaa*lacU169* (*φ80 lacZ*aaaM15) *hsdR17 recA1 endA1 gyrA96 thi-1 relA1*	Takara, Dalian, China
β2155	Transconjugation donor: *thrB1004 pro thi strA hsdS lacZ*Δ*M15 (F’lacZ*Δ*M15lacI^q^ traD36 proA^+^ proB^+^) dap* : : *erm recA* : : *RP4-2-tet* : : *Mu-km bbbpir, Erm^r^ Tet^r^ Kan^r^*	Oswald et al., 1999
**Plasmids**		
pEMOC2	Transconjugation vector: ColE1 *ori mob* RP4 *sacB*, *Amp^r^ Cm^r^*	Baltes et al., 2003
pEΔ*lip40*	Up- and down- stream arms of *lip40* were ligated into pEMOC2, and used as the transconjugation vector for *lip40* gene deletion	This work
pJFF224-XN	*E. coli*–*A. pleuropneumoniae* shuttle vector: RSF1010 replicon; *mob oriV*, *Cm^r^*	Frey, 1992
pJFF-*lip40*	pJFF224-XN carrying the intact *lip40* of *A. pleuropneumoniae* SLW01, and used for the construction of complementation strain	This work
**Primers**		
Lip40-F1	5′-TTGTCGACGAGTTGGAAGAGATTATTGC-3′, forward primer with *Sal*I site (underlined) comprising position -927 to -908 of the *lip40* coding sequence	This work
Lip40-R1	5′-GGTCTAGACTCCTCATAGATATTATAGGCG-3′, reverse primer with *Xba*I site (underlined) comprising position -28 to -7 of the *lip40* coding sequence	This work
Lip40-F2	5′-GGTCTAGACATATTGATTTAATACGCAAAGCG-3′, forward primer with *Xba*I site (underlined) comprising position +898 to +919 of the *lip40* coding sequence	This work
Lip40-R2	5′-TTGCGGCCGCGTCTTGGCATACCAAGCATT-3′, reverse primer with *Not*I site (underlined) comprising position +1953 to +1972 of the *lip40* coding sequence	This work
Lip40-F3	5′-GGCTGCAGATGAAAAACATCACAAAATTTG-3′, upstream primer with *Pst*I site (underlined) comprising position +1 to +22 of *lip40*. This primer was used to clone *lip40* gene for construction of complementation stain	This work
Lip40-R3	5′-TTGCGGCCGCTTACTTTTGTTGTTTTGCGC-3′, downstream primer with *Not*I site (underlined) comprising position +878 to +897 of *lip40*	This work
Lip40-F4	5′-AACCGAAACAAGATCAGCCG-3′, forward primer comprising position +134 to +153 of *lip40*. This primer was used for verification of the transcription of *lip40*	This work
Lip40-R4	5′-CTCCTGCCAGTTCCTTAGCA-3′, reverse primer comprising position +795 to +814 of *lip40*. This primer was used for verification of the transcription of *lip40*	This work
pEM-F	5′-TTTCAGGAGCTAAGGAAG-3′, forward primer was used to confirm the absence of transconjugation plasmid in the *A. pleuropneumoniae* gene-deleted mutant	This work
pEM-R	5′-CACCAATAACTGCCTTAA-3′, reverse primer was used to confirm the *A. pleuropneumoniae* gene-deleted mutant	This work
pJFF-F	5′-GAATTTTACCCGGATTGACC-3′, forward primer was used for identification of the complementation strain by verifying the presence of the shuttle vector in *A. pleuropneumoniae* cells	This work
pJFF-R	5′-GCTGAAACTTTGCCATCGTA-3′, reverse primer was used for identification of the complementation strain	This work
ApxIV-F	5′-CAGAATCAAACTTTCGGCG-3′, forward primer was used to confirm bacterial colonies isolated from mouse lung tissues are *A. pleuropneumoniae*	Schaller et al., 2001
ApxIV-R	5′-GCACAAGGTAAAACGGTGA-3′, reverse primer used confirm bacterial colonies isolated from mouse lung tissues are *A. pleuropneumoniae*	Schaller et al., 2001

### Construction of Mutant Strains

The *lip40* gene was deleted in the genome by polymerase chain reaction (PCR). After digestion with the appropriate restriction enzymes, the two homologous arms were ligated into the transconjugation vector pEMOC2 (Baltes et al., 2003), resulting into a *lip40* knockout vector, pEΔ*lip40*, which was then transformed into the *E. coli* β2155 (Oswald et al., 1999) to generate the donor cells for transconjugation. The donor cells were co-cultured with the *A. pleuropneumoniae* SLW01 for 4 h so as to introduce the plasmid into the recipient cells. After chloramphenicol-mediated positive selection and sucrose-mediated counter-selection, chloramphenicol-sensitive and sucrose-resistant colonies were chosen. The absence of *lip40* in the genome of these colonies was confirmed by PCR and sequencing, and named as Δ*lip40*. For construction of the complementation strain, intact *lip40* gene was cloned and inserted into the *E. coli*–*A. pleuropneumoniae* shuttle vector pJFF224-XN (Frey, 1992), and the resulting complementation plasmid pJFF-*lip40* was transformed into *lip40* deletion mutant by electroporation. Chloramphenicol-resistant transformants were selected and recognized as C*Δlip40*. The presence of *lip40* gene and shuttle vector was verified by PCR. The transcription of *lip40* gene in the SLW01, Δ*lip40* and C*Δlip40* was determined using reverse transcription (RT)-PCR.

### Bacteria–Cell Adherence Assay

The adherence assay was performed using a bacteria–cell model with a monkey origin cell line [formerly known as the St. Jude Porcine Lung Epithelial Line (SJPL)] as described previously (Li et al., 2011). Briefly, *A. pleuropneumoniae* fresh culture at mid-log phase (OD_600_ ∼0.8) was harvested and washed three times with Dulbecco’s modified Eagle’s medium (DMEM; Gibco BRL). Bacteria were incubated with monolayers of SJPL cells in 6-well plates with a multiplicity of infection of ∼100:1 for 2 h. Planktonic and loosely attached bacteria were removed by five washes of DMEM. To measure the number of adherent bacteria per well, cells were resuspended in distilled water and cell-associated bacteria were liberated by repeated pipetting. Bacteria were serially diluted and plated onto TSA agar. After incubation for 24 h at 37°C, colonies were counted.

### Bacterial Colonization in Mouse Lung Tissues

To investigate the role of Lip40 in *in vivo* colonization, Twenty-four 6-week-old female BALB/c mice were purchased from the Center for Disease Control of Hubei Province (Hubei CDC, Wuhan, China), and divided into four groups of six mice each. Groups I, II and III were inoculated intraperitoneally with 10^6^ colony-forming units (CFUs) in 200 μl TSB of *A. pleuropneumoniae* SLW01, Δ*lip40*, or CΔ*lip40*; group IV received TSB in the same manner and served as a negative control. Mice were monitored for 24 h and dying mice were euthanized. Surviving mice were euthanized at 24 h after infection. Lung tissue (0.1 g) was removed under sterile conditions from each mouse and homogenated. Bacteria in the lung homogenates were quantified by serial dilution and plating onto TSA plates. After incubation for 24 h at 37°C, bacterial colonies morphologically similar to those of *A. pleuropneumoniae* were confirmed using PCR with ApxIVA-specific primers (Schaller et al., 2001). All experiments involving live animals were approved by the Animal Care and Use Committee at Central China Normal University. We declare that all animals were treated humanely and in compliance with all applicable institutional animal care guidelines in China.

### Experimental Infection in Pigs

To evaluate the influence of Lip40 on the pathogenicity of *A. pleuropneumoniae*, a pig infection assay was performed as described previously (Liu et al., 2009). Eighteen 7-week-old pigs were purchased from an *A. pleuropneumoniae*-free herd and randomly divided into four groups, with five pigs in groups I, II and III, and three pigs in group IV. *A. pleuropneumoniae* fresh cultures at OD_600_ ∼0.8 were harvest (deduced viable count ∼10^9^ CFU/ml). The cultures were placed on ice and diluted in ice-cold TSB to the desired live cell counts (appropriate 2.5 × 10^7^ CFU for each pig). The viable bacterial counts of these cultures were determined simultaneously by the plate-counting method. Pigs were anesthetized by intravenous injection of ketamine (4 mg/kg) and xylazine (2 mg/kg) before infection. Pigs in groups I, II, and III were injected intratracheally with *A. pleuropneumoniae* SLW01, Δ*lip40*, or CΔ*lip40* suspended in 5 mL TSB, separately. Pigs in group IV received 5 ml TSB and served as a negative control. Clinical signs of pigs were monitored for 7 days post-infection (dpi). Dying pigs with severe dyspnea and low temperature (<38°C) were humanely euthanized. At 0, 6, 12, 24, 36, 48, 60, and 72 hour post-infection (hpi), rectal temperature was measured. Pigs with a temperature >41°C were considered to have high fever. Other clinical signs, such as appetite decrease, breathing difficulty and lethargy were monitored and evaluated as described previously (Fuller et al., 2000). Blood samples were collected every morning before infection and after infection, blood glucose (BGlu) was detected using a kit from Nanjing Jiancheng Biotech, Co., Ltd. (Nanjing, China). At 7 dpi, the surviving pigs were euthanized for postmortem examinations. Lung tissues were excluded carefully and fibrinous adhesion to pleura was recorded. The lung lesion scores were determined as described previously (Hannan et al., 1982). For histological analysis, lung samples were fixed in 10% formalin buffer (pH 7.2). Thin sections (5 μm) were stained using hemotoxylin and eosin and examined by light microscopy.

### Statistical Analysis

The data obtained from the present study were expressed as mean ± SD. Student’s *t*-test was used to compare the differences between two groups for adherence and colonization abilities, clinical signs, BGlu levels and pathological lesions. *P* < 0.05 was considered significant, and *P* < 0.01 were considered highly significant.

## Results

### Generation of Δ*lip40* and CΔ*lip40*

After sucrose counter-selection, these chloramphenicol-sensitive and sucrose-resistant transconjugants were selected and named as Δ*lip40*. During PCR verification, SLW01 showed a long fragment (2917 bp), whereas Δ*lip40* exhibited a short truncated fragment (2020 bp) using primers Lip40-F1 and Lip40-R2 (**Supplementary Figure S1A**), and the mutant was negative in PCR using primers pEM-F and pEM-R (**Supplementary Figure S1A**). CΔ*lip40* was constructed based on Δ*lip40*. The *E. coli*–*A. pleuropneumoniae* shuttle vector pJFF-*lip40* was confirmed to be stable in CΔ*lip40*, when they were passaged in the medium without antibiotic pressure (**Supplementary Figure S1B**). Besides, both SLW01 and CΔ*lip40* were positive in the RT-PCR analysis, whereas no amplicon was obtained from the *lip40* gene deleted mutant Δ*lip40* (**Supplementary Figure S1C**). Growth curves of the *A. pleuropneumoniae* in the TSB medium showed no obvious differences between the WT and mutant strains (**Supplementary Figure S1D**), suggesting that mutation in *lip40* and transformation with pJFF-*lip40* did not affect the growth of *A. pleuropneumoniae*.

### Δ*lip40* Exhibits Reduced Cell Adherence

**FIGURE 1:**
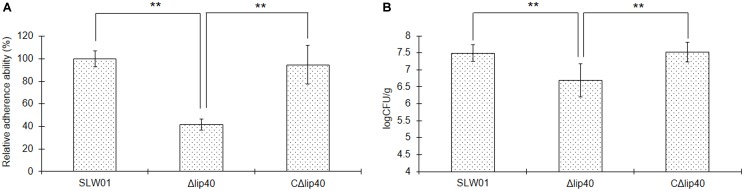
Roles of Lip40 in the *Actinobacillus pleuropneumoniae* Adherence **(A)** and Colonization **(B)**. The adherence assays were performed as described previously (Li et al., 2011). Bacteria adhered to the surface of cells were cultured on TSA plates and CFUs were counted. The adherence assay was repeated at least three times. The percentage CFU was normalized to the WT group and designated as 100%. For the *in vivo* colonization assay, lung tissues were taken under sterile conditions and homogenized. Homogenates were diluted in TSB and plated onto TSA agar. Bacteria loads were calculated according to the colonies on the plates and dilution ratio (CFU/g). Double asterisks represent highly statistically significant differences (*P* < 0.01).

To understand better the roles of Lip40 in *A. pleuropneumoniae* infection, we analyzed the adherence of the *A. pleuropneumoniae* mutant to SJPL cells. Viable bacteria before and after interaction with SJPL cells were grown on the TSA plates and the CFUs were counted. The adherence of Δ*lip40* was significantly lower than that of SLW01 (*P* < 0.01, **Figure 1A**). The decrease in adherence was recovered by trans-complementation, the difference in cell adherence between SLW01 and CΔ*lip40* was not significant (*P* > 0.05, **Figure 1A**). The results indicated that absence of Lip40 could lead to reduced adherence of *A. pleuropneumoniae* to SJPL cells.

### Mutation in *lip40* Affects *in Vivo* Colonization of *A. pleuropneumoniae* in Mice

The cell adhesion assay revealed that mutation of Lip40 significantly decreased the cell adherence of *A. pleuropneumoniae*. To understand better the effects of Lip40 deletion on the virulence-associated phenotypes, an *in vivo* colonization assay was carried out using a mouse infection model. BALB/c mice infected with WT, Δ*lip40* and CΔ*lip40* were monitored intensively for 24 h. Lung tissues of all these mice were removed under sterile conditions and weighed immediately after the animals were euthanized. Lung homogenates were diluted and cultured on TSA plates. The negative control group showed no visible colonies on the plates. The average lung bacterial load of the WT group (3.5 × 10^7^ CFU/g) was ∼4.6-fold more than that of the Δ*lip40* group (7.6 × 10^6^ CFU/g) (**Figure 1B**, *P* < 0.01). The average bacterial load of the CΔ*lip40* group was 3.9 × 10^7^ CFU/g, which was significantly higher than that of the Δ*lip40* group (*P* < 0.01). Results suggest that Lip40 is involved in *in vivo* colonization.

### Deletion in *lip40* Significantly Attenuates Virulence of *A. pleuropneumoniae* in Pigs

The results of the cell adherence and bacterial load assays suggest that Lip40 is related to *A. pleuropneumoniae* infection. Therefore, the role of Lip40 in bacterial virulence was determined using a pig infection model. Pigs inoculated with SLW01 and CΔ*lip40* strains developed typical clinical symptoms of porcine pleuropneumonia, including high temperature, reduced appetite, accelerated breathing, cough and lethargy, whereas pigs infected with Δ*lip40* mutant showed milder signs. Clinical sign scores of pigs were monitored and summarized in **Table 2**. Rectal temperature of pigs at eight time points after infection was monitored. Pigs in the negative control group had normal temperature. The infected pigs showed elevated temperature at 6 hpi; temperature in the Δ*lip40*-infected group gradually recovered from 12 hpi and returned to normal at 24 hpi; in the SLW01- and CΔ*lip40*-infected groups, fever remained and recovered after 48 hpi. Pigs in groups I and III exhibited reduced willingness to take food, and more food was left in their hoppers; the appetite indexes were significantly higher than those of the animals infected with Δ*lip40* (*P* < 0.05) and in the negative control group (*P* < 0.01) (**Table 2**). Pigs infected with SLW01 and CΔ*lip40* moved slowly or laid down; the lethargy indexes of pigs in these groups were significantly higher than those of the Δ*lip40* and control groups (*P* < 0.01) (**Table 2**). Pigs in groups I and III exhibited significantly higher dyspnea indexes than those of the Δ*lip40* and control groups (*P* < 0.01) (**Table 2**). One pig in the SLW01 infected group developed severe symptoms at 3 dpi and was euthanized; other pigs were alive during the observation period. These results indicated that mutation in *lip40* dramatically reduced the ability of *A. pleuropneumoniae* to cause clinical signs in pigs.

**Table 2:** Clinical signs and lung lesions of pigs in different infection groups.

Strains	Dose administrated (CFU)	Appetite^a^	Dyspnea^a^	Lethargy^a^	Fever^b^	Lung lesions^c^
SLW01	2.5 × 10^7^	2.2 ± 0.8^d^	1.9 ± 0.6^e^	2.0 ± 0.4^d^	19/40	17.2 ± 4.0^e^
Δ*lip40*	2.5 × 10^7^	0.8 ± 0.4	0.7 ± 0.5	0.6 ± 0.3	6/40	3.3 ± 1.6
CΔ*lip40*	2.5 × 10^7^	2.0 ± 0.7^d^	1.7 ± 0.3^e^	1.8 ± 0.4^e^	20/40	13.8 ± 2.8^e^
Negative control	0	0	0	0	0/24	0

^a^*Clinical signs, such as appetite, dyspnea and lethargy, were evaluated as described previously (Fulleretal., 2000). Appetite: score 0, did eat; score 1, did not eat; and total score = number of 12-h periods not eating over 36 h observation period; dyspnea and lethargy were scored as: 0, normal; 1, slight; 2, moderate; 3, severe. Results presented as mean ± SD. ^b^Rectal temperature was monitored at 0, 6, 12, 24, 36, 48, 60 and 72 hpi, temperature >41ˆ∘C was considered a high fever. High fever/total observations were recorded. ^c^The lung lesion score was assessed as described previously (Hannanetal., 1982). A complete lung was divided into seven lobes and each was arbitrarily allotted a maximum possible lesion score of 5. Lesions of each lobe were assessed and recorded as a fraction of 5, and the lung lesion score was calculated as the sum of the seven lobes. ^d^Significant difference compared with the Δ*lip40*-infected group (*P* < 0.05). ^e^*P*<0.01 compared with the Δ*lip40*-inoculated group*.

**FIGURE 2:**
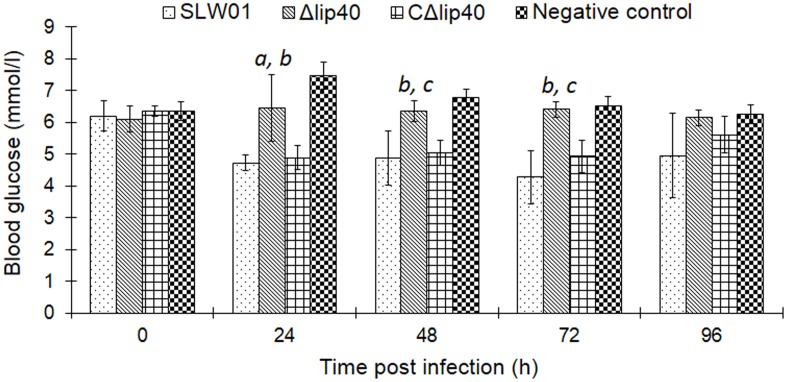
Detection of Blood Glucose (BGlu) in Pigs Inoculated with *A. pleuropneumoniae*. BGlu were evaluated using detection kits produced by Nanjing Jiancheng Biotech, Co., Ltd. (Nanjing, China). *^a^*Significant difference between the SLW01-infected group (*P* < 0.05); *^b^P* < 0.01 compared with the CΔ*lip40*-infected group; *^c^P* < 0.01 compared with the SLW01-infected group.

Blood glucose was determined every morning before and after infection. Δ*lip40*-infected animals showed slight but not significant alteration of BGlu at 24 hpi, relative to that in the negative control group (**Figure 2**). BGlu levels in pigs in the SLW01- and CΔ*lip40*-infected groups dropped significantly at 24 hpi, compared to those in the Δ*lip40*-infected group (*P* < 0.01). The BGlu level of a seriously diseased pig in the SLW01-infected group was only half the normal level at 72 hpi, and this pig was then euthanized for ethical reasons. These results revealed that the mutation in *lip40* alleviates changes in BGlu caused by *A. pleuropneumoniae* infection.

**FIGURE 3:**
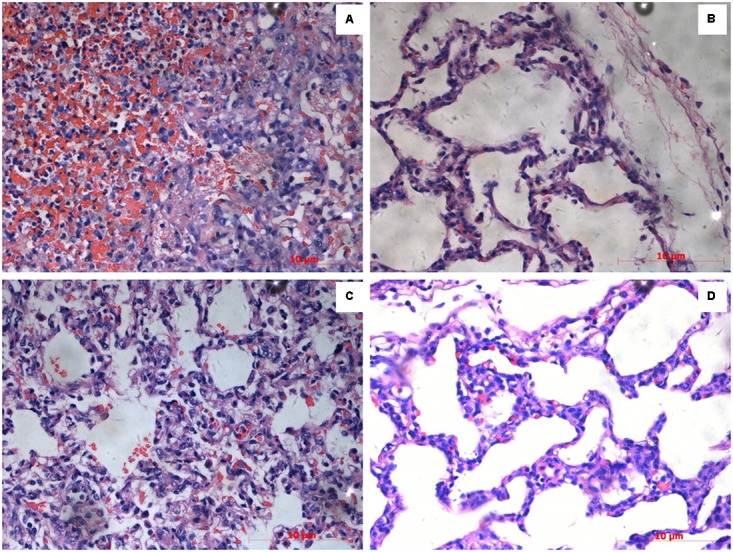
Histopathological examinations. Pigs were inoculated with *A. pleuropneumoniae* WT, Δ*lip40*, CΔ*lip40* or TSB (from **A–D**). Scale bar at the lower right corner of each picture represents 10 μm. **(A)** Numerous inflammatory cells, erythrocytes and exudations were observed in the alveoli of pigs infected with WT. **(B)** Lung sections of Δ*lip40*-infected pigs exhibited slightly thickened pleura and alveolar walls. **(C)** Presence of erythrocytes in alveoli indicated hemorrhagic pneumonia in the CΔ*lip40*-inoculated pigs. **(D)** Normal lung tissue of the TSB control group.

The lung tissues in the SLW01 and CΔ*lip40* groups were red with several dark areas, and showed a rough pleural surface. In contrast, the lungs of Δ*lip40*-infected pigs were pink with no obvious changes. SLW01 and CΔ*lip40* induced significantly higher lung lesion scores than Δ*lip40* did (*P* < 0.01) (**Table 2**). The lung sections from the SLW01- and CΔ*lip40*-infected groups were different from those from Δ*lip40*-infected pigs. A mass of inflammatory cells, erythrocytes and exudations were observed in the alveoli of pigs in the SLW01 and CΔ*lip40* groups (**Figure 3**), suggesting that these pigs had hemorrhagic pneumonia. Pigs infected with Δ*lip40* exhibited normal lung sections except for the presence of slightly thickened pleura and alveolar walls (**Figure 3**). These results indicated that Lip40 contributed significantly to the virulence of *A. pleuropneumoniae*.

## Discussion

Rapid attachment to the respiratory tract and efficient adaptation to the challenging *in vivo* environment are required for the development of bacterial disease. Many lipoproteins have been found to contribute to these processes, and are considered to be involved in the pathogenicity of bacteria of public health importance (Becker and Sander, 2016), as well as pathogens of veterinary importance (Hatfaludi et al., 2010). *A. pleuropneumoniae* is one of the most important swine pathogens causing severe damage to the respiratory system. Involvement of lipoproteins in the virulence of *A. pleuropneumoniae* has been reported previously. The transferrin-binding protein TbpB is important for bacterial virulence and enables *A. pleuropneumoniae* to utilize porcine transferrin (Baltes et al., 2002). Besides, a recent study indicated that another virulence-associated lipoprotein VacJ of *A. pleuropneumoniae* was implicated in outer membrane integrity maintenance, biofilm formation, and complement resistance (Xie et al., 2016). These findings revealed the significance of lipoproteins in *A. pleuropneumoniae* infection. However, sixty lipoproteins have been predicted from *A. pleuropneumoniae* genome (Hu et al., 2015), only few lipoproteins have been investigated. Therefore, more studies are still needed to gain better understanding of the roles of *A. pleuropneumoniae* lipoproteins.

Lip40 of *A. pleuropneumoniae* was annotated as a hypothetical protein during genome sequencing analysis (Xu et al., 2008). It was presumed to be required for the virulence of *A. pleuropneumoniae* (Hu et al., 2015). In this study, we confirmed that Lip40 mediated adhesion of the bacteria to SJPL cells. Although the SJPL cell line is of monkey origin (Silversides et al., 2010), it is useful for investigating *A. pleuropneumoniae*–cell interactions (Auger et al., 2009; Li et al., 2011). Cell adherence is recognized as one of the most important factors that determines the entry of pathogenic bacteria into the host, and is associated with the virulence and pathogenicity (Stones and Krachler, 2015). Several factors, like ApfA and Adh, have been shown to mediate adherence of *A. pleuropneumoniae* to host cells, vaccination with these adhesins provided effective protection against lethal *A. pleuropneumoniae* challenge in murine models (Zhou et al., 2013; Wang et al., 2015). Besides, mutation in the coding sequences of these adhesins reduced *A. pleuropneumoniae* virulence to animals (Zhou et al., 2013; Wang et al., 2015). Our previous study demonstrated that Lip40 is an immunoprotective agent (Hu et al., 2015), together with the present result, indicating the importance of this adhesin in *A. pleuropneumoniae* infection. Moreover, Lip40 was not only involved in adhesion to host cells, but also found to be required for *in vivo* colonization in this study. This inferred that the failure of *in vivo* proliferation may be partially due to the reduced capacity of bacteria–cell adherence. Colonization is critical for the virulence of pathogenic bacteria (Siegel and Weiser, 2015). Previous studies have revealed that mutation in *A. pleuropneumoniae* potential virulence factors TolC2, PdxS/PdxT, and SapA results in reduced *in vivo* colonization, as well as attenuated virulence (Li et al., 2017; Xie et al., 2017a,b). Therefore, it is reasonable to speculate that Lip40 plays an important role in the virulence of *A. pleuropneumoniae*, by mediating bacterial adherence and colonization.

*Actinobacillus pleuropneumoniae* is considered to be an important pathogen that has pigs as its natural host, despite several studies indicating that mice can be used for *A. pleuropneumoniae* immunization and infection assays (Zhou et al., 2013). Therefore, a pig infection model was used to confirm whether Lip40 contributes to the virulence of *A. pleuropneumoniae*. The pig infection model has been commonly used to investigate relevant genes involved in the pathogenesis of *A. pleuropneumoniae* infection and evaluate the efficacy of vaccines against *A. pleuropneumoniae* (Hannan et al., 1982; Fuller et al., 2000; Liu et al., 2009). A set of clinical sign indexes, postmortem examination as well as histological analysis, make the assessment of porcine pleuropneumonia efficient and reliable. In this study, we found that pigs in the Δ*lip40*-infected group developed less clinical symptoms, much milder necrotic lung lesions and histopathological lesions, relative to those of the SLW01-infected group. Trans-complementation of the mutant restored these virulence-associated phenotypes. Additionally, we found that the BGlu levels of the WT and CΔ*lip40* groups were lower than those of the Δ*lip40* group, at 24 and 48 hpi. The decreased appetite may have been partly responsible for the drop in BGlu levels. Besides, hypoglycemia was considered as an indicator of bacterial infection, and associated with high mortality during pneumococcal infections (Jan et al., 2009). Here, we observed that the BGlu level of one pig in the SLW01-infected group dropped to half the normal level at 72 hpi; this pig was in poor condition and died soon after the biopsy, this pig showed extensive pleural adhesion and necrotic lung lesions in the subsequent postmortem examination, indicating that it had severe pleuropneumonia. Taken together, these results confirmed that Lip40 was required for the infection of *A. pleuropneumoniae*.

## Conclusion

The roles of Lip40 protein in the pathogenesis of *A. pleuropneumoniae* have been investigated in the present study. Our results suggest that Lip40 protein is involved in bacterial cell adhesion. We also illustrated that Lip40 contributes to *in vivo* colonization. The *A. pleuropneumoniae* Lip40 knockout strain exhibited reduced virulence in the pig infection model. Further investigations of the potential of Lip40 protein as part of a subunit vaccine would be valuable for the prevention of *A. pleuropneumoniae* infection, and studies focusing on the Lip40–host cell interaction and structural features of Lip40 protein may provide new insight into the pathogenesis of *A. pleuropneumoniae*.

## Author Contributions

CQ and QG designed the research and provided experiment conditions. JL and CQ wrote the paper. JL, YC, LG, and LZ executed the experiments. DY, JZ, and JM contributed to the animal experiments. SG, JY, and HZ helped with the data analysis. All authors reviewed and approved the manuscript.

## Conflict of Interest Statement

The authors declare that the research was conducted in the absence of any commercial or financial relationships that could be construed as a potential conflict of interest.

## Supplementary Material

FIGURE S1 Verification of *Actinobacillus pleuropneumoniae* mutants and growth curves. **(A)** PCR verification of the *A. pleuropneumoniae* gene-deleted mutant primer pairs Lip40-F1/R2 and pEM-F/R. Lane M, DNA ladder DL15000 (Takara, Dalian, China); lane 1, WT; lane 2, single cross-over transconjugant; lanes 3 to 6, Δ*lip40*. **(B)** PCR analysis of the complementation strain CΔ*lip40* using primers pJF-F and pJF-R. Lane M, DNA ladder DL2000; lanes 1 to 10, CΔ*lip40* of different passages; lane 11, positive control (plasmid pJFF-*lip40*); lane 12, negative control. **(C)** RT-PCR confirmation. RNA samples were extracted from *A. pleuropneumoniae* WT, Δ*lip40* and CΔ*lip40* cultures and reverse-transcribed into cDNA, separately. The cDNA was used as template in the subsequently PCR analysis with primers Lip40-F4 and Lip40-R4. Lane M, DNA ladder DL2000; lane 1, WT; lane 2, Δ*lip40*; lane 3, CΔ*lip40*; lane 4, negative control. **(D)** Growth curves of the *A. pleuropneumoniae* WT, Δ*lip40* and CΔ*lip40*. The growth curves were obtained from the average of at least three repeats.
