# Supplementary figures and images for: Outer Membrane Lipoprotein Lip40 Modulates Adherence, Colonization, and Virulence of Actinobacillus pleuropneumoniae
Source: Front Microbiol. 2018 Jul 3;9:1472. doi: 10.3389/fmicb.2018.01472 (PMC6038445; doi:10.3389/fmicb.2018.01472)

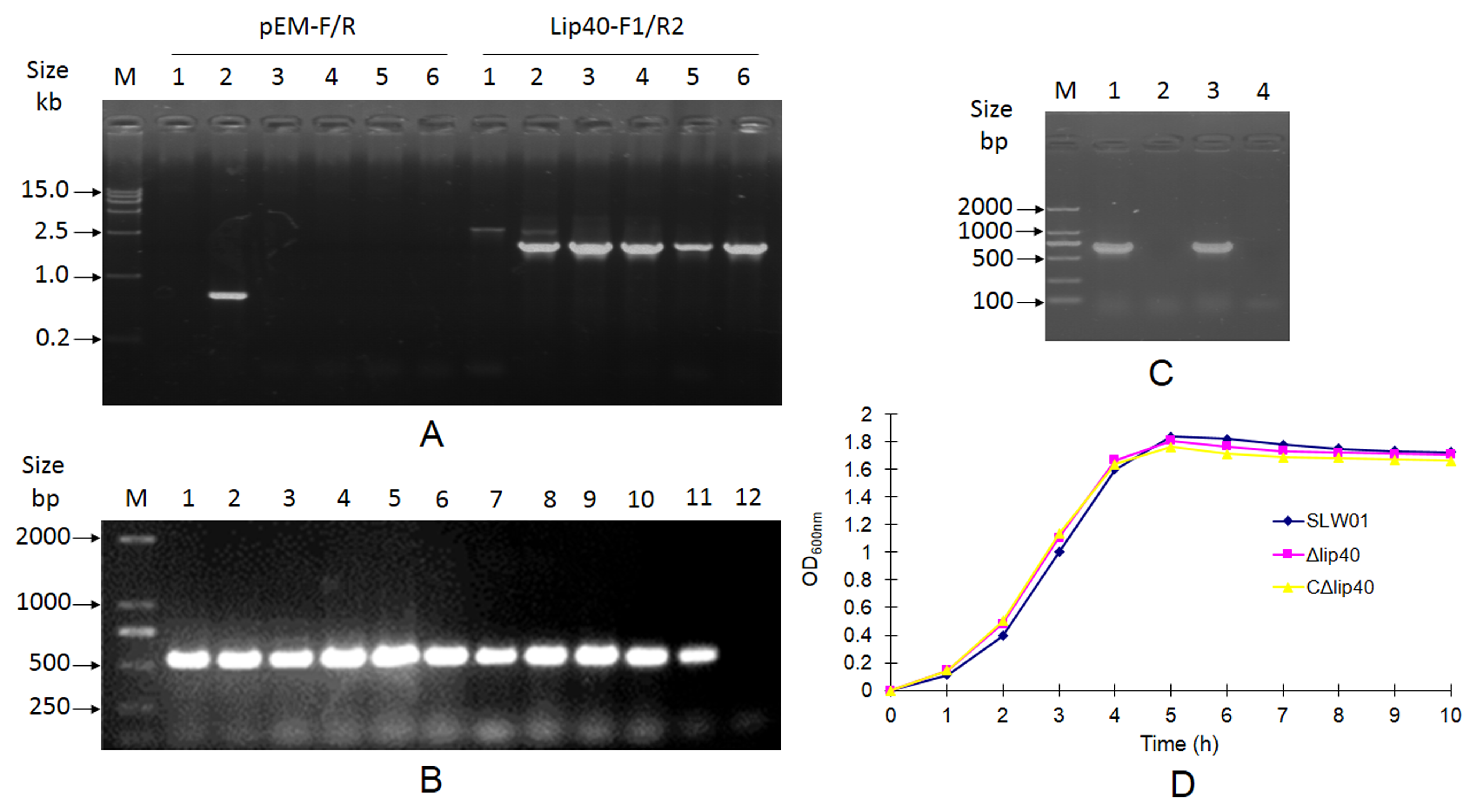

Supplement: FIGURE S1 — Verification of Actinobacillus pleuropneumoniae mutants and growth curves. (A) PCR verification of the A. pleuropneumoniae gene-deleted mutant primer pairs Lip40-F1/R2 and pEM-F/R. Lane M, DNA ladder DL15000 (Takara, Dalian, China); lane 1, WT; lane 2, single cross-over transconjugant; lanes 3 to 6, Δlip40. (B) PCR analysis of the complementation strain CΔlip40 using primers pJF-F and pJF-R. Lane M, DNA ladder DL2000; lanes 1 to 10, CΔlip40 of different passages; lane 11, positive control (plasmid pJFF-lip40); lane 12, negative control. (C) RT-PCR confirmation. RNA samples were extracted from A. pleuropneumoniae WT, Δlip40 and CΔlip40 cultures and reverse-transcribed into cDNA, separately. The cDNA was used as template in the subsequently PCR analysis with primers Lip40-F4 and Lip40-R4. Lane M, DNA ladder DL2000; lane 1, WT; lane 2, Δlip40; lane 3, CΔlip40; lane 4, negative control. (D) Growth curves of the A. pleuropneumoniae WT, Δlip40 and CΔlip40. The growth curves were obtained from the average of at least three repeats. [file Image_1.TIF]
